# Operating Ethically: A Review of Surgical Ethics in Pakistan and Recommendations for the Way Forward

**DOI:** 10.7759/cureus.46789

**Published:** 2023-10-10

**Authors:** Maheen Zakaria, Russell Seth Martins, Mohammad Umair Khan, Asad Saulat Fatimi, Baila Maqbool, Saulat Hasnain Fatimi

**Affiliations:** 1 Medical College, Aga Khan University Medical College, Karachi, PAK; 2 Thoracic Surgery, Hackensack Meridian Health, New Jersey, USA; 3 Department of Acute Care Surgery, University of New Mexico School of Medicine, Albuquerque, USA; 4 Department of Cardiothoracic Surgery, Aga Khan University Hospital, Karachi, PAK

**Keywords:** informed consent, ethical recommendations, bioethics recommendations, surgery, bioethics

## Abstract

Medical ethics underpin the moral framework that delineates the professional relationship between physicians and their patients and thereby is an integral part of making patient-centric healthcare decisions. The concept of ethics is deeply embedded in the field of surgery as surgeons confront a myriad of dilemmas as a part of their routine, whether it be in a preoperative or postoperative environment. The current review aims to describe the state of surgical ethics in Pakistan, with the intent of encouraging dialogues about the ethical considerations relevant to the field surgery that will identify actionable areas for improvement. While most surgeons are aware of the traditional principles of ethics and their practice, their surgical and clinical decisions may fall short of these standards because of time constraints and prevailing cultural and religious beliefs and taboos. The rigorous application of ethical principles in areas of patient-related communication, such as consenting, trainee education, palliative and end-of-life care, and surgical innovation and research, will have significant implications for patients, surgeons, and society. Our review has identified the lack of formal bioethics education and insufficient oversight and ethical regulations to be at the core of inadequate ethical practices in Pakistan and has highlighted actionable areas to be addressed in the future.

## Introduction and background

Derived from the Greek word “ethos” meaning character and the Latin word “mores” meaning customs [1], the word “ethics” refers to a system of moral principles that govern human behavior, decision-making, and ways of living one’s life. Given the degree of human interaction that accompanies the medical profession, as well as the responsibility to provide the best possible care to patients at their most physically and emotionally vulnerable, the field of medicine is inevitably fraught with ethical dilemmas. Surgical ethics are particularly challenging due to the invasive nature and procedural risks associated with the specialty, with surgeons routinely encountering ethical concerns while obtaining informed consent, counseling anxious patients, delivering disappointing news, and dealing with quality-of-life (QoL) concerns [2].

A self-evident tenant acknowledged by educators and practitioners at all levels of the medical profession anywhere in the world is that there should not be country-specific ethical standards by which human beings receive healthcare: one set of standards should apply to all. However, while traditional ethical principles encompass respect for autonomy, beneficence, non-maleficence, and justice, a context-specific understanding of ethical principles, especially in lower- and middle-income countries (LMICs) in South Asia, such as Pakistan, where factors including religion and cultural practices may significantly impact ethical discourse, is crucial to implementing ethical practices with adequate rigor. Furthermore, these factors may cause the ethics discourse and approach in Pakistan to differ from that of high-income countries (HICs) [3].

We believe that due to a lack of formal bioethics education in undergraduate and postgraduate training programs, insufficient oversight of surgical practice by governmental and professional regulatory bodies, and the limited avenues that are available for legal redress of patient-perceived lapses practice or behavior, ethical standards for surgeons in Pakistan are not meeting those of their counterparts in most HICs [3]. In light of this gap, the recognition of the need to improve and formalize the training of surgeons and physicians in Pakistan with regard to medical and surgical ethics is growing [4].

It is important to note that a context-specific understanding of surgical ethics is imperative for an equitable surgical healthcare system in Pakistan, and such a system is unachievable with technical competence and implementation alone [2]. This narrative review aims to discuss and explore the current landscape of surgical ethics in Pakistan and highlight actionable areas to be addressed in the future.

## Review

Informed consent

Informed consent refers to voluntary, uncoerced agreement of a person to undergo a treatment after they have a clear understanding of the whole and truthful facts, implications, and consequences associated with the procedure [5]. The concept of informed consent also applies to surgical research and disclosure of patient health information (PHI). Informed consent forms the foundation of the trust between surgeon and patient and ensures compliance with the ethical principles of autonomy and beneficence. Despite these noble aspirations, informed consent proceedings are often considered a “mere formality” by physicians and surgeons in Pakistan [6,7], thereby leading to significant inconsistencies in the integrity and effectiveness of the process [7]. Consent is frequently taken just a day before, and not uncommonly on the day of, or even minutes before the operation itself [8,9], thereby depriving patients of adequate time for deliberating, reflecting, or asking questions pertaining to their treatment. Moreover, in Pakistan, consent is usually obtained by a junior, non-operating team member, and sometimes even by paramedical staff, instead of the operating surgeon themselves [10,11]. This problem is particularly prevalent in public hospitals [6]. In some rural hospitals, consent-taking is omitted entirely in approximately 10% of cases and is grossly unstandardized and inadequate, thereby reflecting poor prioritization of this most basic and critical ethical measure in surgery [10].

Meanwhile, given literacy disparities in Pakistan [12], it is undoubtedly a mammoth challenge to standardize the informed consent process while ensuring complete comprehension by the patient. Even in tertiary care hospitals with established informed consent protocols, nearly two-thirds of patients report not understanding the information provided to them [6]. Reasons for this include the complexity and inaccessibility of commonly used medical terminologies, language barriers and cultural differences, or consent being taken by persons insufficiently acquainted with procedure details [6,9,10]. Half of the patients may not even read the consent form before signing, and many are reluctant to ask important questions due to the inherent power imbalance and paternalistic dynamic between the provider and the patient. To exacerbate matters, patients are largely unaware of their rights and are content with the minimal information that is disclosed to them, thereby allowing healthcare institutions to continue using improper or inadequate informed consent practices without consequences or sanctions [6,9]. Furthermore, it is often not even the patients themselves who are responsible for their medical decisions; in Pakistan, up to 70-90% of patients opt to relinquish their right to provide informed consent, preferring family, friends, or even the surgeon to assume the decision-making role [13,14]. Often, this decision lies in the hands of the family member who are responsible for the hospital bill [15]. The practice of surrogate consent can be attributed to the collectivist nature of Pakistani family and social culture [8], which, though socially acceptable, becomes an ethical issue when the patients’ desires conflict with those of the surrogate.

Transparency and communication

Ethical communication is predicated on certain values, such as honesty, and being responsible with one’s words and for the actions that may be a consequence of those words. It is of utmost concern that many physicians in Pakistan consider it unnecessary to explain the details of their treatment plans to their patients [16] given the information, or lack thereof, would significantly impact the patients’ decision-making process. Amin et al. reported that at one university hospital in Pakistan, nearly 70% of patients reported not being informed of potential complications of surgical procedures [10]. Moreover, surgeons may be more likely to discuss the consequences of not pursuing the surgery with patients than they are to discuss possible complications, possibly because surgeons feel knowledge of these potential complications may deter patients from pursuing what may be a life-saving procedure. Although it is understandable that surgeons may want to spare their patients undue anxiety, no matter how well-intentioned, this glaring lack of transparency in communication violates patients’ right to information about their medical treatment and stems from the deep-seated paternalistic attitudes within the medical profession in Pakistan. In addition, surgeons are often unclear about what constitutes a breach of patient confidentiality and privacy, which may have negative psychological impacts on patients [7,10]. It is extremely important that surgeons dedicate time to explaining the consequences of any procedure to their patient, especially in the context of the coronavirus disease 2019 (COVID-19), which has brought about new risks, such as hospital-acquired COVID-19 pneumonia and associated hospital course, as a part of the standard informed consent process [17].

Posthumous organ donation

Posthumous organ donation and procurement is one aspect of surgical ethics in which many countries around the world have settled for an opt-out approach based on presumed consent, as this increases the availability of life-saving organ transplant surgeries. However, although it is highly unlikely that patients in Pakistan discuss posthumous organ donation with their family and provider, presuming permission to procure organs because of lack of pre-mortem documentation indicating refusal violates the inherent criteria for ethical consent [18]. Ashraf et al. found that a large proportion of Muslims feel that Islam, the predominant religion in Pakistan, does not permit organ donation, and that almost 40% of people are unwilling to donate their organs [19]. In such a context, organ donation based on presumed consent may have the effect of disturbing the relationship between the provider and patients’ family members. It also contributes to creating among the patient and their family the suspicion that providers have a vested interest in not saving the lives of critically ill patients, particularly those from a low socioeconomic background [18]. Thus, in Pakistan, the more culturally appropriate and ethical course of action would be to seek uncoerced permission from patients and their family members. Moreover, since most of the major religions in Pakistan (Islam, Christianity, and Hinduism) support the individual’s decision to donate organs [20], it would be in accordance with the prevailing religious climate of the country to involve spiritual leaders in educational efforts regarding posthumous organ donation.

Surgeon-patient relationship

It is important to realize the profound impact of culture on the dynamics of the surgeon-patient relationship to understand the differences between this relationship in different parts of the world. In HICs around the world, this relationship is progressing toward a more patient-centric, egalitarian collaboration [21], while in LMICs like Pakistan, this transition has been met with resistance, and the doctor-patient relationship remains more paternalistic in nature [22]. This may be attributable to several factors. Medical professionals may have socially constructed attitudes of superiority over other professions, perhaps because only the most academically talented high school students become doctors in Pakistan [23], which may lead to surgeons disregarding opinions of the patient who, in Pakistan, is most likely to be uneducated. In addition, in Pakistan, physicians are widely regarded as standing “next to God” or as father-like figures, resulting in patients following their surgeons’ advice unreservedly [24]. This paternalistic practice of medicine is exacerbated by the fact that there is no oversight and evaluation with regard to whether ethical principles have been followed in most patient encounters in Pakistan, which increases the risk of unethical behaviors, especially against those of lower socioeconomic strata, such as concealing diagnosis of serious illnesses, and violating patient confidentiality to disclose PHI [24]. In addition, in the fast-paced surgical setting where time-sensitive procedures are involved, surgeons may be more inclined to assume a paternalistic attitude toward patient care [25].

Ethics in surgical education

Although the clinical teaching of medical students and surgical trainees is a surgeon’s obligation in teaching hospitals, patients may not be comfortable or perhaps even be unaware that aspects of invasive procedures will be performed by a person other than the surgeon themselves [6]. Oftentimes, it is presumed that patients consenting to surgical healthcare at a teaching hospital implicitly agree to the hands-on involvement of students and trainees in their operative procedures. Even if such a clause is stated in the patients’ informed consent forms, consent forms are usually signed without being read or without the patient having a comprehensive understanding of the content of the document being signed as mentioned previously [6]. However, this is an affront to patients’ autonomy, as they owe no moral obligation to participate in the training of future surgeons [26]. Surgeons are obligated to meticulously evaluate and supervise their trainees, along with disclosing their role to the patient verbally and on the consent form, hence balancing both their educational and ethical responsibilities. However, in Pakistan, large numbers of patients in understaffed centers may lead to greater inadequately supervised involvement or unsatisfactory clinical training of trainees in surgical healthcare [27], compromising patient rights and also potentially health outcomes. This is more likely to happen to patients from marginalized populations, such as drug users, older patients, and those from low socioeconomic backgrounds [26].

Currently, both the Pakistan Medical Commission (PMC) and the Higher Education Commission (HEC) recommend the inclusion of bioethics in the undergraduate medical curriculum. The wisdom of this recommendation has been questioned by some surgeons, since surgical trainees in Pakistan acquire most of their knowledge of ethics indirectly through observation of senior surgeons [28]. However, many trainees in Pakistan have expressed dissatisfaction with the quality of education in medical ethics, and many have judged the practice of their senior colleagues’ as unethical [28,29]. Witnessing such unethical behavior can adversely and permanently influence trainees’ own perceptions of ethics. Thus, a formal ethics curriculum in surgical training and medical school and ongoing ethics education for surgeons throughout their careers should be a pressing current priority, as it would make ensure future surgeons conform to higher ethical standards and thereby ascribe to a more patient-centric model of care [30], which would not otherwise be possible without a thorough understanding of ethical principles and their implementation in surgical practice.

Palliative and end-of-life care

Restricting discussion of surgical outcomes to solely clinical parameters, such as length of survival, while neglecting patients’ QoL issues incentivizes surgeons to prioritize prolonging life regardless of patient satisfaction post-operatively and ignores consideration of the integration of palliative care into their comprehensive care [31]. Noteworthy in this regard is the fact that, in comparison to other specialties, critically ill patients under the care of surgical services, on average, have poorer QoL [32]. The American College of Surgeons emphasizes the critical importance of catering to patients’ palliative care needs, stressing that even patients with a good likelihood of recovery may benefit from palliative care consultations [33]. Surgical palliative care in the West has been shown to improve outcomes, “generate cost-savings,” and reduce the length of stay in the ICU [34,35]. Currently, palliative care is not a component of undergraduate or postgraduate training in Pakistan [36]. Owing to the lack of openness around the topic of death, end-of-life care discussions are rare in Pakistan [15], and surgeons may lack the ability or inclination to discuss openly and with compassion patients’ poor prognosis and their long-term expectations and aspirations [37]. Surgeons’ attitudes toward palliative care are far from standardized and may be influenced by their own ethos and religious beliefs and those of their patients’ and caregivers, as well as medicolegal issues and implications [38]. Other challenges to palliative care in Pakistan include a scarcity of systems and institutions to handle chronic pain and a lack of institutional prioritization of palliative care [15,39]. To improve the state of surgical palliative care in Pakistan, it is crucial that national and institutional stakeholders commit to bringing widespread reform in palliative care policy, education, and practice, which will ultimately contribute to better patient satisfaction, QoL, patient-doctor relationships, and long-term outcomes.

Surgical research and innovation

Innovation is the driving force behind progress in surgical fields, and such an innovation is usually directed at improving operative techniques, reducing surgical times, and minimizing the cost of operations. In this era of rapidly advancing technology, surgical innovations are likely to advance with ever-increasing speed, and complex and novel surgical procedures may indeed be lifesaving for otherwise untreatable conditions. However, to facilitate an accelerated rate of novel developments in the surgical field, more stringent and rigorous ethical guidelines are necessary, without which patients may be put at risk since minimizing risks and maximizing benefits and safety are of paramount priority [40]. Strict, well-implemented guidelines and knowledge to deal with the inevitable ethical issues associated with research are currently lacking in Pakistan, both in theory and in practice, which consequently leads to stagnation in surgical innovations and a restriction in the scope of surgical research to observational-based studies.

One example of such an ethical issue is that pertaining to informed consent. All matters of informed consent, as discussed above, apply in full to patient recruitment into clinical and surgical trials since surgeons and researchers are required to provide and discuss with patients the information they must know and comprehend before agreeing to participate in any study, including the possible known and unknown outcomes of the procedure. This is in accordance with the recommendations of the IDEAL (Idea, Development, Exploration, Assessment, and Long-Term Study) Collaboration to plan surgical trials and evaluations of novel techniques more effectively and safely [41]. Informed consent is even more crucial for surgical trials since surgeons are obliged to inform their patients of the fact that they may be performing a procedure for the first time and divulge to the patient their training, experience, and qualification to conduct the novel operation [42,43]. It is thus vital that surgeons operating as a part of surgical trials possess not only sufficient technical expertise and experience but also a comprehensive understanding of the ethical implications of surgical research to qualify the surgeons’ inclusion in the trial and to ensure patients’ safety and the best possible outcomes.

Recommendations and solutions

Given the current landscape of surgical ethics in Pakistan, we would like to summarize our recommendations regarding improvement of ethical practices for the future follows:

1. A publicly available and easily accessible national ethics standard with comprehensive and context-specific guidelines should be drafted, especially with regard to matters of informed consent, transparency in communication, posthumous organ donation, patient-centric attitudes toward patient care, formal bioethics education, and surgical innovations and research. To the best of our knowledge, the last such guideline was drafted more than two decades ago by the Pakistan Medical and Dental Council (PMDC) in 2002.

2. Undergraduate medical education should emphasize the importance of medical ethics and an adequate formal bioethics education should be integrated into the curriculums of medical colleges across Pakistan. Furthermore, postgraduate surgical training should re-emphasize and revisit these concepts throughout the program. Adequate understanding and implementation of ethical principles in patient interactions can also be made a core competency of surgical residency programs. Proven mechanisms that integrate clinical care and ethics, such as ethics grand rounds or morbidity and mortality meetings, must be developed and routinely implemented.

3. Mechanisms of oversight should be established not only at the institutional level at all medical institutions in Pakistan but also at the national and governmental level to ensure that recommendations (1) and (2) are being adhered to faithfully.

4. Well-developed ethics questions should be included as a requirement in national certification exams for medical students and residents graduating from surgical (or non-surgical) residencies.

5. Verbal and written discourse around the field of surgical ethics should be increased by increasing the coverage of ethics-related topics at institutional and national surgical conferences. For example, one of the main learning objectives for the 7th Aga Khan University Annual Surgical Conference was to “address the ethical approach to surgical research by using scientific data and reports.”

6. Public awareness campaigns could be conducted so as to educate the general public regarding their rights as patients, particularly the right to participate in the healthcare decision-making process.

## Conclusions

The safe and equitable practice of surgery requires a balance of technical expertise and ethical reasoning. This review broadly touches upon the ethical issues that have persisted for years in surgery and require immediate remedy. Formal bioethics education in the undergraduate medical curriculum and postgraduate training programs is grossly inadequate, which is a deficit that may account for the significant ethical lapses of currently active physicians and surgeons in Pakistan. There is a need for the government and medical professional societies to work on the state of surgical ethics in Pakistan by looking into improved informed consent practices and policies that prioritize patient autonomy, a partnership patient-care model to replace the existing paternalistic one, increased awareness among patients about their rights, formal ethics training for aspiring and practicing surgeons, a focus on palliative care to improve patient satisfaction, and greater supervision over surgical research and innovation. To improve the the quality of surgical care being offered to patients in Pakistan and portray the surgical field as one that prioritizes humanistic care, investing time and resources into the teaching of ethics and the adequate implementation and monitoring of ethical standards and practices is necessary.
